# Effects of Gut Microbiota on Hypertension and the Cardiovascular System

**DOI:** 10.3390/nu15214633

**Published:** 2023-10-31

**Authors:** Daotong Li, Fang Chen

**Affiliations:** National Engineering Research Center for Fruit and Vegetable Processing, Key Laboratory of Fruits and Vegetables Processing, College of Food Science and Nutritional Engineering, Ministry of Agriculture, Engineering Research Centre for Fruits and Vegetables Processing, Ministry of Education, China Agricultural University, Beijing 100083, China; lidaotong@cau.edu.cn

Cardiovascular diseases, which include hypertension and atherosclerosis, are a group of disorders that affect the heart and blood vessels. They are among the leading causes of mortality worldwide, and 17.9 million people die from cardiovascular diseases each year, accounting for about a third of global deaths. Notably, most deaths from cardiovascular diseases occur in low- and middle-income countries [1]. According to data from the American Heart Association, the annual direct and indirect costs of cardiovascular disease deaths total more than USD 316.1 billion [2]. Although the pathogenesis of cardiovascular disease is not fully understood, genetic and environmental factors are known to be involved in the development of these disorders. A known environmental risk factor for cardiovascular disease development is poor dietary habits, which have been reported to account for about 10 million deaths of non-communicable diseases worldwide [3]. Thus, it is imperative to develop effective dietary strategies to prevent the development of cardiovascular disease.

The human gastrointestinal tract is inhabited by a large microbial community, which is referred to as the gut microbiota. It is composed of approximately 100 trillion microbes, including bacteria, archaea, and fungi [4]. The gut microbiota is essential for human health and numerous functional features of the gut microbiome, such as digesting dietary polysaccharides, metabolizing xenobiotic drugs, promoting immune system responses, and protecting against pathogen invasion [5]. Over the past decade, it has become clear that gut microbiota plays a vital role in the development of metabolic diseases, including cardiovascular diseases. The correlation between cardiovascular diseases and gut microbiome has been suggested in many studies. For instance, patients with atherosclerotic stroke have an altered gut microbiota characterized by an increased abundance of opportunistic pathogens, such as *Enterobacter*, *Oscillibacter*, and *Desulfovibrio*, and a decreased abundance of beneficial bacteria, such as *Bacteroides*, *Prevotella*, and *Faecalibacterium* [6]. The gut microbiota composition was changed in patients with heart failure with the significant depletion of short-chain fatty acid-producing bacteria [7].

The evidence supporting the causal role of gut microbiota in cardiovascular disease development has also been revealed. Diet, as one of the most important factors shaping the gut microbiome, has been shown to play a key role in the progression of cardiovascular disease. For instance, the dietary supplementation of mice with choline and betaine was able to promote atherosclerosis via the regulation of the macrophage scavenger [8]. Mechanically, the gut microbial metabolism of phosphatidylcholine is an important step contributing to the pathogenesis of cardiovascular disease [8]. Dietary L-carnitine, a nutrient in red meat, can be metabolized by intestinal microbiota to produce trimethylamine and trimethylamine-N-oxide, which are important gut flora-derived metabolites linked to the risk of cardiovascular disease risk [9]. Choline diet-induced trimethylamine-N-oxide production and atherosclerosis susceptibility can be transferable to germ-free mice through fecal microbial transplantation [10]. Furthermore, the targeted inhibition of trimethylamine production by microbial inhibitors is able to alleviate atherosclerotic lesion development [11]. Therefore, elucidating the precise interrelationships between diet and gut microbiota can guide novel microbiome-based preventative and therapeutic strategies for cardiovascular disease.

Hypertension, which is also known as high blood pressure, is the most important risk factor for cardiovascular disease. Over the past few decades, the number of people with hypertension has markedly increased, and it is estimated that one-third of adults are hypertensive, which contributes to 10.8 million global deaths [12]. The etiology of hypertension is complex and has not been elucidated. Emerging evidence suggests that the interplay of both genetic and environmental risk factors is involved in the pathogenesis of hypertension [13]. Recently, data from a genome-wide association study provided evidence that identified hypertension-associated loci, which explains 27% of heritability [14]. A growing body of evidence has emerged supporting a potential role for gut microbiota dysbiosis in the development of hypertension. A significant decrease in microbial richness, diversity, and evenness, as well as an increased *Firmicutes*/*Bacteroidetes* ratio, has also been detected in hypertensive animals and patients [15]. Similarly, in a cohort of 196 Chinese participants, a decrease in microbial richness and diversity was observed in both pre-hypertensive and hypertensive populations when compared to healthy controls [16]. Pre-hypertensive and hypertensive patients harbored a *Prevotella*-dominated enterotype, while the healthy controls had a *Bacteroides*-dominated enterotype. Moreover, an overgrowth of Klebsiella has been detected in hypertensive populations. Notably, elevated blood pressure can be transferrable through fecal microbiota transplantation [16]. Many lines of evidence from animal studies also demonstrate a link between gut microbiome and hypertension. Germ-free mice, in which the gut microbiota is absent, display lower blood pressure when compared to conventional mice [17]. Consistently, angiotensin II–induced vascular dysfunction and hypertension are mitigated in germ-free mice [18]. Of note, blood pressure and vascular contractility can be restored through the introduction of the gut microbiota to germ-free rats, further confirming that blood pressure can be modulated by the gut microbiota [19]. Trimethylamine N-oxide, the metabolite generated from the metabolism of dietary choline by the gut microbiota, has also been demonstrated to aggravate angiotensin II–induced hypertension [20]. In this Special Issue, the study by Chen and colleagues [21] examines plasma metabolite profiles and their relationships to oral/gut microbiota in a cross-sectional cohort involving 52 hypertensive participants and 24 healthy controls. Significant differences were found in plasma metabolites between the hypertensive participants and participants without hypertension. Importantly, both the oral and gut microbial community composition had significant correlations with the metabolites related to the regulation of blood pressure. 

Overall, these findings highlight the pivotal role of gut microbiota and their metabolites as key factors for the cause of high blood pressure. The manipulation of the gut microbiome may represent a new strategy for the prevention of hypertension.
